# Evaluating Communication Performance in Rotating Electrical Machines Using RSSI Measurements and Artificial Intelligence

**DOI:** 10.3390/s24248209

**Published:** 2024-12-23

**Authors:** Sonia Ben Brahim, Samia Dardouri, Hanen Lajnef, Amel Ben Slimane, Ridha Bouallegue, Tan-Hoa Vuong

**Affiliations:** 1 InnoV’COM Laboratory-Sup’Com, University of Carthage, Ariana 2083, Tunisia; hanen.lajnef@supcom.tn (H.L.); ridha.bouallegue@supcom.tn (R.B.); 2 Department of Computer Science, College of Computing and Information Technology, Shaqra University, Shaqra 11911, Saudi Arabia; 3 Faculty of Computing and Information, Al Baha University, Al Baha 65526, Saudi Arabia; aslimane@bu.edu.sa; 4 LAPLACE Laboratory-UMR5213, National Polytechnic Institute of Toulouse, 31077 Toulouse, France; tan-hoa.vuong@enseeiht.fr

**Keywords:** RSSI, artificial intelligence, rotating electrical machines

## Abstract

This paper introduces a novel methodology for evaluating communication performance in rotating electric machines using Received Signal Strength Indication (RSSI) measurements coupled with artificial intelligence. The proposed approach focuses on assessing the quality of wireless signals in the complex, dynamic environment inside these machines, where factors like reflections, metallic surfaces, and rotational movements can significantly impact communication. RSSI is used as a key parameter to monitor real-time signal behavior, enabling a detailed analysis of communication reliability. The methodology comprises several stages, including data collection, preprocessing, feature extraction, and model training. Various machine learning models are implemented and evaluated. Among these, the SVM model with a Radial Basis Function (RBF) kernel outperforms others, achieving an accuracy of 97%, with high precision and recall scores, confirming its robustness in classifying RSSI data and handling complex signal behavior. The confusion matrix further supports the SVM model’s accuracy, showing minimal misclassification.

## 1. Introduction

Rotating electrical machines are indispensable in various industrial sectors, including manufacturing, energy production, and transportation [1]. These machines play a pivotal role in converting electrical energy into mechanical energy, or vice versa, forming the backbone of critical operations across power plants, factories, and transport systems [2]. Given their essential functions, ensuring the reliable and efficient operation of rotating machines is paramount, especially in high-stakes environments where even minor failures can lead to costly downtime and potential safety risks [3]. Therefore, real-time monitoring of these machines is a key factor in enhancing performance, reliability, and longevity.

Wireless communication has emerged as a promising solution for the real-time monitoring of rotating machinery due to its flexibility and ease of installation [4]. However, ensuring stable and accurate wireless communication within a rotating machine is a significant challenge. The rotation of the antenna introduces Doppler effects, and the electromagnetic interference from the machine’s operation further complicates signal propagation [5]. Additionally, the machine’s internal components, particularly metal parts, can reflect, scatter, and absorb the wireless signals, leading to fluctuations in communication performance [6]. Despite these challenges, it remains crucial to ensure reliable wireless communication, as any communication disruption can undermine the real-time monitoring system’s effectiveness, leading to missed signals or inaccurate data. Given the complexity of the environment inside rotating electrical machines, studying the performance of wireless communication in real-time becomes highly relevant. To effectively assess and ensure the communication link’s integrity, the Received Signal Strength Indication (RSSI) is a valuable metric [7]. RSSI measures the power of the received signal and provides insights into the quality and reliability of the wireless communication link within the machine [8]. Monitoring the RSSI in real time allows for the detection of signal degradation due to environmental factors, helping to evaluate whether the communication is robust or likely to fail. However, simply measuring the RSSI in real time may not be enough to provide a comprehensive evaluation of communication performance. To enhance the analysis and offer a more nuanced understanding of the communication quality, artificial intelligence (AI) techniques can be applied [9]. By incorporating AI, it is possible to categorize the wireless communication as either “Good” or “Bad” based on predefined criteria, enabling more advanced control and monitoring systems. By applying machine learning algorithms, AI can classify communication quality more accurately and predict potential failures before they occur [10,11]. This advanced approach not only enhances decision-making but also leads to the development of intelligent monitoring systems that autonomously adapt to changing conditions, ensuring that communication remains robust and reliable despite the machine’s dynamic environment.

Our study presents a novel contribution by leveraging RSSI as a practical and responsive indicator of signal quality, enhanced by AI-based models that identify complex patterns in communication fluctuations under rotational conditions. RSSI serves this purpose effectively due to its sensitivity to environmental changes; combined with AI algorithms, it provides real-time insights into signal reliability and anticipates potential disruptions. This integrated approach addresses existing challenges, offering a scalable and adaptive solution tailored specifically for rotating electrical machines.

In this paper, an innovative methodology is proposed that combines real-time wireless communication performance evaluation with artificial intelligence to address the unique challenges presented by rotating electrical machines. The methodology follows four key stages: (1) real-time data collection through an advanced hardware setup to capture RSSI and monitor communication link strength, (2) data preprocessing and feature extraction to prepare the collected data for analysis, (3) training several machine learning models to classify the RSSI data, and (4) evaluating the models to identify the most effective approach. The machine learning algorithms tested include five methods: Dummy Classifier, Gaussian Naive Bayes, Logistic Regression, Quadratic Discriminant Analysis (QDA), and Support Vector Machines (SVM) with both Radial Basis Function (RBF) and polynomial kernels. Each model is trained and tested to categorize RSSI data into classes reflecting communication quality within the machine.

Through performance comparison, this study identifies the optimal approach for reliable, real-time communication monitoring in such a complex environment. This methodology marks a significant contribution in overcoming the challenges of wireless communication in rotating electrical machines, creating new avenues for enhanced system control and monitoring through AI integration. This preliminary investigation assesses RSSI-based communication performance within a rotating machine environment under controlled experimental conditions. This study serves as a foundational step toward understanding RSSI’s potential to indicate communication quality, with further validation across varied setups necessary for broader applicability.

This article is structured into five main sections, each addressing a key component of the research. The second section, related work, provides an overview of existing studies. The third section, description of the proposed methodology, outlines the innovative approach developed in this study, which includes four distinct phases: data collection through advanced hardware setups, data preprocessing and feature extraction, machine learning model training, and model evaluation. In the results and discussions section, the performance of the machine learning models is thoroughly analyzed. Finally, the conclusions section summarizes the contributions of this work and outlines future directions for research.

## 2. Related Works

The monitoring and control of electrical machines have traditionally relied on older methodologies, such as mathematical modeling and analytical approaches [12]. Early work in this field focused on using mathematical equations to predict machine performance and diagnose faults. For example, classical methods like the state–space representation, differential equations, and equivalent circuits were commonly employed to model the dynamic behavior of rotating electrical machines [13]. While these models provided valuable insights, they often required extensive manual calculations and could be limited in their ability to adapt to rapidly changing conditions in real-time applications [14]. In recent years, wireless communication systems have emerged as a promising tool for the online monitoring and control of rotating electrical machines. One such notable work is [6]. The system employed wireless sensors to collect data such as temperature and vibration, enabling real-time condition monitoring without the need for extensive wiring. This approach demonstrated the practicality of wireless technologies in industrial environments, reducing setup complexity and improving scalability. However, the focus was not on assessing the performance of wireless communication itself, such as evaluating the signal strength and reliability under varying machine conditions.

Received Signal Strength Indicator (RSSI) is a key metric in wireless communication, widely used to assess the quality of a wireless signal. Though research on applying RSSI specifically within rotating electrical machines is scarce, its importance in general telecommunication systems is well documented. In wireless sensor networks (WSNs) and Internet of Things (IoT) applications, RSSI is often used to evaluate link quality, optimize network performance, and even estimate distances between devices [15]. Studies such as [16] highlight how RSSI can be crucial in environments with physical obstructions or interference, providing insights into how it can be used to improve communication reliability in challenging settings. The evaluation of RSSI in these scenarios serves as a foundation for its potential application in more complex environments, such as inside rotating machines.

Artificial Intelligence (AI) has increasingly been integrated into performance evaluation systems across various domains [17,18]. In general, AI techniques such as machine learning (ML) and deep learning have proven to be highly effective in predicting system performance, identifying patterns, and diagnosing issues [19]. One notable example is [20], which applied AI for the real-time performance evaluation of complex communication networks. Their research utilized deep learning models to predict network performance degradation based on signal quality metrics, similar to RSSI, providing real-time adaptive solutions to optimize the communication infrastructure. Such approaches underscore how AI can be a powerful tool for real-time performance evaluation and predictive maintenance in various systems, including wireless communication setups in industrial environments.

Another significant application of AI and machine learning is in Tire Pressure Monitoring Systems (TPMS) [21]. The work [22] showed how AI models could significantly improve the reliability of TPMS, allowing for predictive maintenance and reducing the risk of tire failure during operation. This serves as an example of how machine learning can be employed in performance monitoring for dynamic, real-time systems. The integration of AI in TPMS underscores the potential for similar techniques to be applied to the monitoring of rotating electrical machines, where real-time data analysis and predictive capabilities can significantly improve system performance and fault detection [23,24].

Moreover, ref. [25] discusses the integration of ML and IoT technologies in industrial applications, particularly within Industry 4.0. This work emphasizes how these technologies improve manufacturing processes through predictive maintenance, security enhancements, process control, and additive manufacturing. The study provides a comparative analysis of IoT architectures and ML strategies, highlighting their effectiveness in optimizing resource management, operational safety, and cost reduction. These findings underline the potential of ML and IoT to bridge the gap between academic research and industrial applications. In summary, while classical models have laid the groundwork for understanding electrical machines, advancements in wireless technology and AI offer new opportunities for more efficient, real-time monitoring systems. These technologies not only reduce manual effort but also provide more precise and adaptive control, making them indispensable for the future of electrical machine monitoring. Table 1 provides a comparison of the discussed methodologies, highlighting the similarities and differences with our approach, which combines RSSI measurements and machine learning models to classify communication quality in rotating electric machines.

## 3. Description of the Proposed Methodology

The proposed methodology for evaluating communication performance within rotating electrical machines represents an innovative integration of real-time RSSI measurements and artificial intelligence, aiming to provide an advanced framework for assessing transmission quality in challenging industrial environments. The methodology unfolds in four essential phases:

- RSSI Measurement Methodology: The first step is a comprehensive approach to accurately capture RSSI values inside and outside the machine, leveraging sophisticated experimental setups to ensure precise and dynamic signal strength data collection.
- Data Preprocessing: The second phase involves the thorough processing of collected data. This step includes binary classification of RSSI values into “Good” or “Bad” based on a predetermined threshold, alongside feature extraction, feature selection, and correlation analysis to refine the dataset. This results in a well-curated dataset that enhances the efficiency of subsequent analyses.
- Model Training: The third stage centers on training predictive models to classify the RSSI data. Utilizing a variety of machine learning algorithms, including Dummy Classifier, Gaussian Naive Bayes, Logistic Regression, Quadratic Discriminant Analysis, and Support Vector Machines (SVM), the dataset is used to create robust models capable of categorizing the communication quality within the machine.
- Testing and Evaluation: The final phase focuses on evaluating the trained models using key performance metrics such as precision, recall, F1-score, error, support, time, and the confusion matrix. This ensures the classification accuracy is validated, providing a reliable means to assess the communication performance.

To enhance clarity, a flow diagram is provided, illustrating the sequential steps of the experimental and analytical process:

1. Start: Indicate the beginning of the process.
2. RSSI Measurement:
  - Action: Collect RSSI values inside and outside the machine.
  - Details: Ensure precise measurements using a sophisticated setup designed for dynamic environments.
3. Data Preprocessing:
  - Action: Process collected RSSI data.
  - Sub-steps:
    ■ Binary classification of RSSI into “Good” or “Bad”.
    ■ Perform feature extraction.
    ■ Conduct feature selection and correlation analysis.
  - Output: Generate a well-refined dataset.
4. Model Training:
  - Action: Train models using machine learning algorithms.
  - Details: Utilize various machine learning algorithms (e.g., Dummy Classifier, Gaussian Naive Bayes, Logistic Regression, Quadratic Discriminant Analysis, Support Vector Machines).
  - Output: Develop robust models capable of accurately classifying communication quality.
5. Testing and Evaluation:
  - Action: Evaluate trained models.
  - Sub-steps:
    ■ Calculate key performance metrics (precision, recall, F1-score, error, support, time).
    ■ Generate confusion matrix.
  - Output: Validate model performance.
6. End: Concludes the methodology.

**Figure 1:**
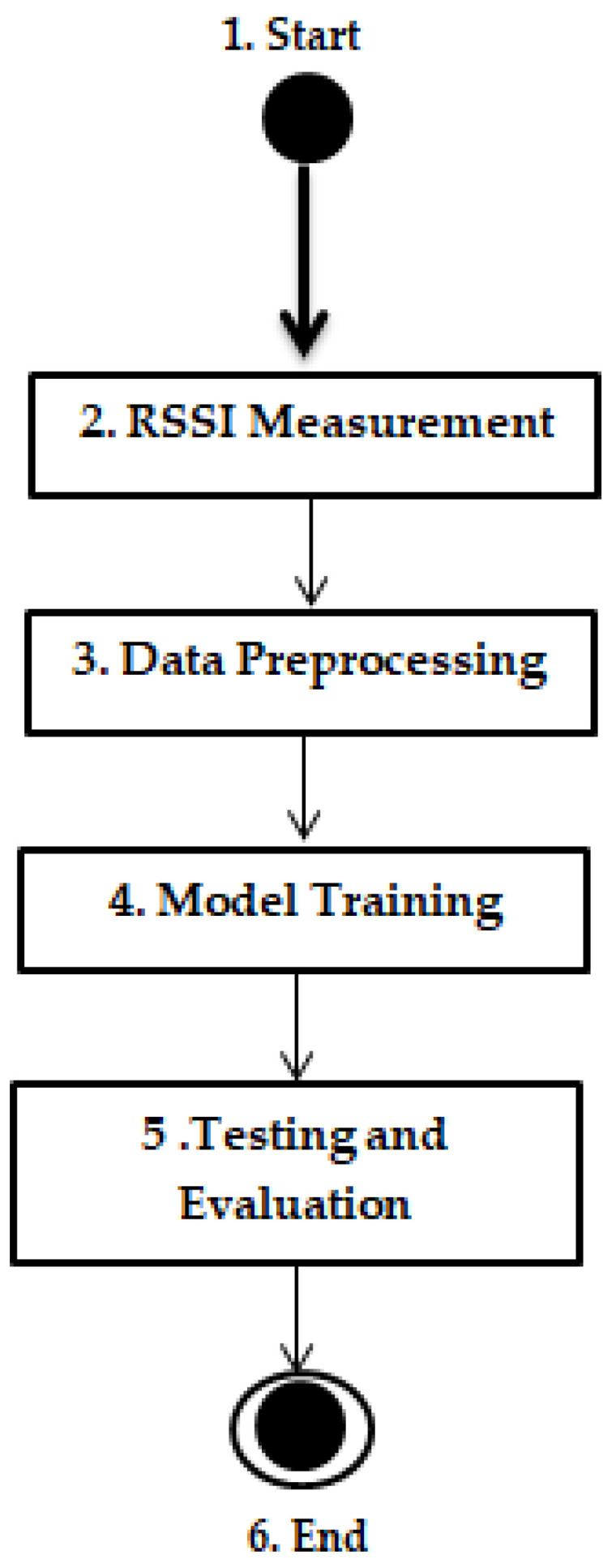
The flowchart diagram of the proposed methodology.

Figure 1 depicts the flowchart diagram of the proposed methodology.

The fusion of artificial intelligence with real-time RSSI measurements offers a powerful tool for monitoring and controlling rotating electrical machines, making this approach valuable for future advancements in industrial automation and predictive maintenance. By effectively characterizing the quality of wireless communication within the machine, this methodology not only enhances reliability but also contributes to the broader field of smart industrial system monitoring, with the potential for applications in machine health assessment, fault detection, and efficiency optimization.

**Figure 2:**
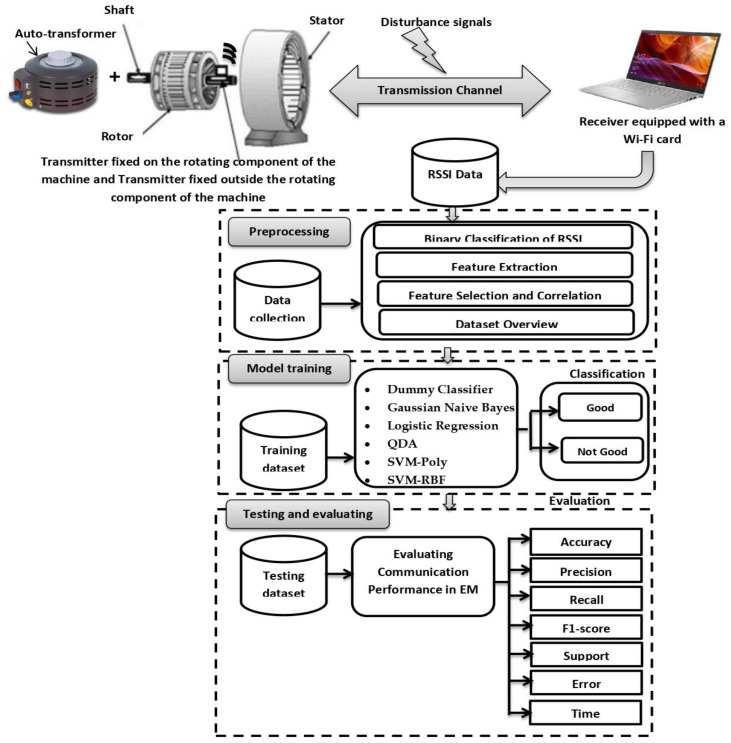
Proposed model architecture.

Figure 2 illustrates the proposed model architecture.

### 3.1. Dynamic Methodology for RSSI Measurement in Rotating Electric Machines

The propagation characteristics of electromagnetic waves within a rotating electric machine are notably complex, requiring an advanced and dynamic solution. This innovative approach focuses on validating these propagation phenomena through real-time signature analysis. The core of the methodology lies in capturing and analyzing the Received Signal Strength Indication (RSSI), which provides a powerful metric for understanding signal behavior. RSSI was specifically chosen for its ability to quantify the signal quality in real time, offering crucial insights into how the signal attenuates and propagates through the machine. This is especially useful in such challenging environments, where factors like metallic surfaces and rotating components significantly influence electromagnetic wave behavior. By monitoring RSSI, the communication link’s performance can be evaluated effectively, making it an ideal metric for assessing wireless communication reliability.

To further examine the environmental impact inside the rotating electric machine on wireless communication, ambient Wi-Fi signal measurements were collected across all 2.4 GHz channels, both inside and outside the machine. Results indicate that interference levels within the machine are generally higher, likely due to metal structures and electromagnetic noise from the machine’s components. Specifically, signal strength inside the machine was observed to be lower across all channels, with noticeable fluctuations aligned with the machine’s rotational movements. These findings highlight the inherent challenges in maintaining reliable communication in such environments, emphasizing the need for robust models that account for these environmental variables.

**Figure 3:**
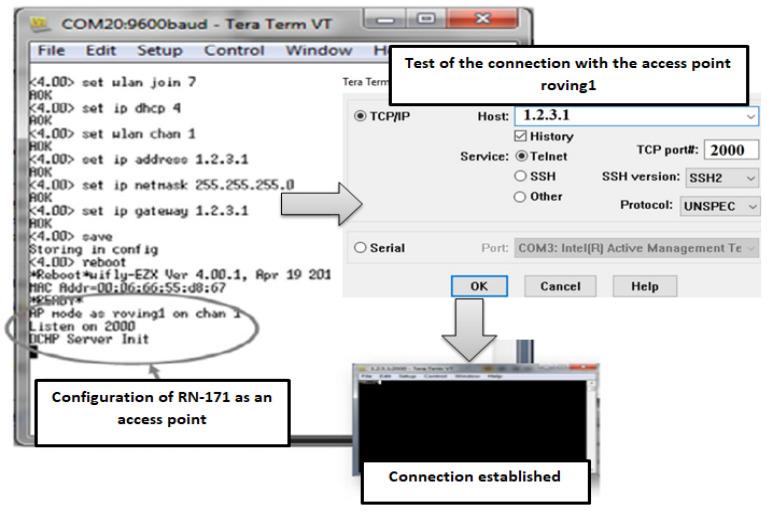
Configuring the RN-171 as an access point.

For this purpose, a continuous wave signal was transmitted from a rotating transmitter to a receiving antenna operating at 2.4 GHz. The hardware setup revolves around the highly efficient RN-171 transmission module, chosen for its innovation in low power consumption and compact design—ideal for mobile wireless applications in this complex environment. The RN-171 module boasts a 32-bit SPARC processor, 2.4 GHz radio, central processing unit (CPU), analog sensor interfaces, real-time clock, power management, TCP/IP stack, and a crypto accelerator, making it a versatile tool for the experimental needs. The RN-171 operates in two primary modes: data transfer mode, where it functions as a data channel, and control mode, allowing configuration through ASCII commands. For this experiment, the RN-171 was configured to operate as an access point to collect real-time RSSI data. Figure 3 demonstrates the setup and configuration of the RN-171 module using the TeraTerm interface [5].

To evaluate wireless communication performance within such a challenging environment, a series of experiments were conducted to measure RSSI both inside and outside the machine at different speeds. In the first experiment, an RN-171 wireless transmitter, operating on the 802.11 standard [26], was placed inside the machine. In the second experiment, the transmitter was positioned outside the machine under the same conditions. In both cases, the machine’s speed was varied using an autotransformer, allowing for four different voltage levels: 0 V, 25 V, 50 V, and 100 V. A PC was used to calculate and log the RSSI values in real-time. The machine employed for this setup was a single-phase asynchronous squirrel cage machine, with its parameters listed in Table 2.

**Figure 4:**
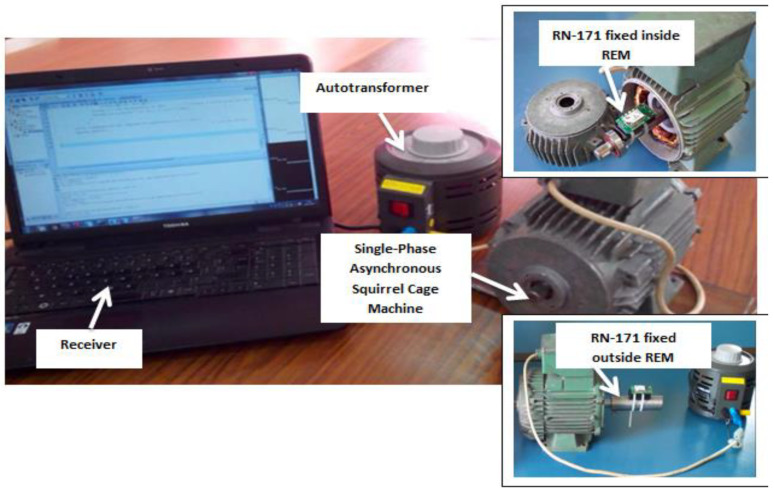
Measurement setup.

Figure 4 illustrates the experimental setup used for these measurements.

This methodology enabled a systematic assessment of communication performance under varying operational speeds and environmental conditions.

**Figure 5:**
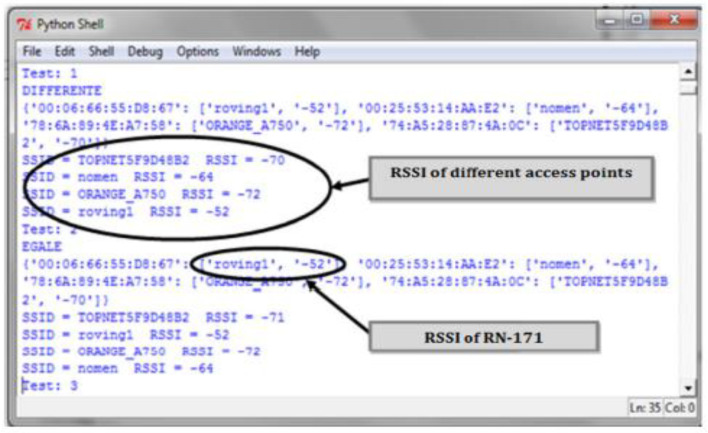
Recovery RSSI of different access points in python.

Figure 5 displays the RSSI values for various access points, as recorded and visualized using the Python programming environment.

This novel integration of the RN-171 module with dynamic signature analysis presents an innovative approach to exploring the complexities of electromagnetic wave propagation within a rotating electric machine, making a significant contribution to signal processing and industrial automation research. RSSI measurements were taken over a period of 100 s for each speed setting: 0 V, 25 V, 50 V, and 100 V with speed variations controlled using an autotransformer.

Measurements were conducted both inside and outside the machine, resulting in a structured dataset comprising four columns: time (t), RSSI values (rssi), speed (u), and measurement location indicator (io), where “0” represents measurements taken inside and “1” represents measurements taken outside. This dataset contains 100 samples for each speed and location, capturing the propagation behavior comprehensively under different operating conditions. The final dataset consists of 800 samples, derived from 100 measurements for each of the four speed settings, taken at two different locations (inside and outside the machine).

**Figure 6:**
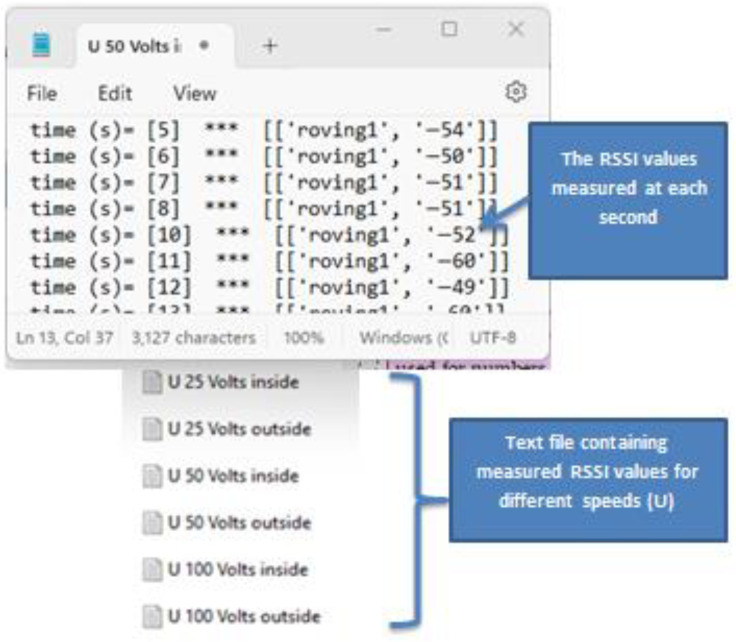
Measured RSSI values for each test case.

Figure 6 illustrates the organization of the recorded RSSI values, showcasing the detailed and systematic collection of data for each test scenario.

### 3.2. Data Preprocessing

Effective data preprocessing was a crucial step in this study to ensure the quality and reliability of RSSI measurements in the rotating electrical machine environment. Given the unique challenges associated with signal noise and potential outliers due to environmental factors, several preprocessing techniques were applied. These steps are described in detail below to enhance the replicability of the study.

#### 3.2.1. Noise Reduction

- Moving Average Smoothing: To mitigate short-term fluctuations and reduce noise in the RSSI signal data, a moving average smoothing technique was applied. This method averages consecutive RSSI readings within a specified window, helping to smooth out rapid, minor variations that are unlikely to represent significant signal changes.
- Low-Pass Filtering: A low-pass filter was also applied to further reduce high-frequency noise while preserving meaningful signal trends. This filtering was particularly beneficial given the rotating machine’s variable interference, helping to isolate genuine signal patterns from transient disturbances.

#### 3.2.2. Outlier Detection and Handling

- Z-score Analysis: Outliers were detected using Z-score analysis, which measures how far a data point is from the mean in terms of standard deviations. RSSI values with Z-scores exceeding a certain threshold (e.g., |Z| > 3) were considered potential outliers. This approach was effective for identifying unusually high or low RSSI values that could skew the classification.
- Winsorization: To handle identified outliers without removing them entirely, Winsorization was employed. This technique limits extreme values by capping them at the nearest threshold within the acceptable range, which preserved the dataset’s integrity while reducing the influence of outlier values.

#### 3.2.3. Data Normalization

- Min–Max Scaling: Since the RSSI values varied significantly due to environmental factors, Min–Max scaling was applied to normalize the data within a range of 0 to 1. This step ensured that all RSSI values were on a consistent scale, facilitating model training and improving the interpretability of classification results.

#### 3.2.4. Handling Missing Data

- Interpolation for Missing Values: Occasionally, data points were missing due to temporary connectivity issues within the machine environment. Linear interpolation was used to estimate and fill these missing values based on surrounding data points, helping to maintain continuity in the dataset.

By employing these preprocessing techniques, the dataset was effectively prepared for analysis, minimizing noise and outlier influence while ensuring data integrity. Each step was tailored to the rotating machine’s specific signal characteristics, thereby enhancing the replicability of this study’s approach.

#### 3.2.5. RSSI Threshold Justification

In this study, a threshold of −50 dB was established to classify RSSI values as either “Good” or “Bad” for communication performance within the rotating electrical machine environment. This selection was carefully determined based on both the specific characteristics of the experimental setup and initial empirical observations. Unlike conventional network environments, where signal strength typically ranges between −40 and −80 dB, the interior of a rotating electrical machine presents unique challenges that influenced the threshold choice.

1. Environmental Characteristics and Challenges
  The interior of a rotating electrical machine creates a complex environment for wireless signals due to several key factors:
    - Interference from Metallic Components: The machine’s structure comprises metallic components that reflect and scatter radio waves, leading to signal degradation and unpredictable fluctuations in RSSI values.
    - Rotational Impact on Signal Stability: As the machine rotates, variations in speed and positioning further contribute to signal inconsistencies. These dynamics mean that a signal considered “strong” or “weak” in a static network setting may behave differently in this mobile, interference-prone environment.
    - Reduced Signal Penetration and Variability: Signals within this enclosed, metallic environment experience reduced penetration and increased variability compared to open-air or standard network environments.

Given these conditions, setting a threshold that could account for environmental influences and identify functional communication levels was essential.

2. Empirical Basis for the −50 dB Threshold
  The selection of −50 dB as a boundary for classifying RSSI values was informed by preliminary testing and analysis. During initial trials, RSSI values in this range were associated with a marked difference in communication performance:
    - Signal Reliability and Usability: An RSSI threshold of −50 dB consistently indicated an adequate signal strength for stable communication in this environment, while values below this threshold correlated with increased packet loss and communication failures.
    - Clear Differentiation in Performance: Preliminary experiments showed that −50 dB served as a clear boundary between reliable and unreliable signal conditions in the rotating machine context. This threshold effectively differentiated “usable” signal quality from weak, inconsistent signals that impeded communication.
3. Comparison with Conventional Network RSSI Ranges
  It is acknowledged that in standard network environments, typical RSSI values range between −40 and −80 dB, with values below −90 dB generally considered extremely weak. However, due to the unique challenges presented within the rotating machine, a threshold adapted to this specific setting was necessary.
    - Adaptation to Environmental Requirements: A higher threshold, such as −50 dB, was selected to ensure that signal quality met the operational requirements of this challenging environment. While standard networks may perform adequately at lower thresholds, the complex nature of this machine setup required a more conservative boundary to maintain reliable communication.

In summary, the −50 dB threshold was determined as the optimal point for distinguishing between “Good” and “Bad” RSSI values within the rotating machine environment. This selection is based on observed signal performance in the experimental setup and reflects the unique demands of this environment, where interference and rotation introduce substantial variability not typically encountered in conventional network deployments.

While RSSI is a widely used metric for evaluating wireless signal strength, it is important to note that its interpretation can vary depending on the chipset and manufacturer. The IEEE 802.11 standard allows for RSSI values in a range from 0 to 255, but each manufacturer defines its own maximum RSSI value (RSSI_Max), which can lead to discrepancies in the scaling and accuracy of RSSI measurements across different devices. In this study, we used the RN-171 module from Microchip for the RSSI measurements. While this module provided consistent and reliable data for our experiments, future research could benefit from incorporating multiple chipsets to assess the impact of chipset-specific differences in RSSI values on communication performance. Such a comparison could offer a more generalizable understanding of wireless communication reliability in complex environments, such as rotating electric machines.

#### 3.2.6. Binary Classification of RSSI

**Figure 7:**
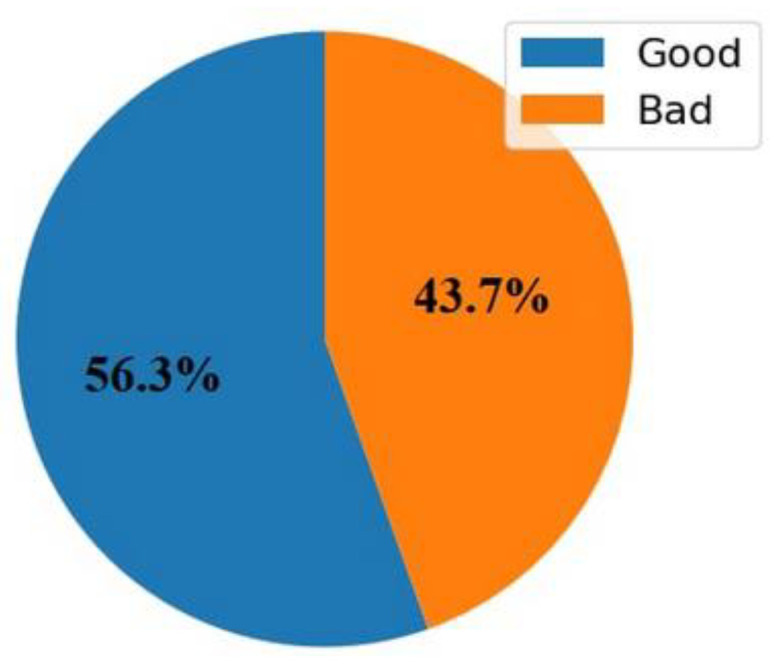
The pie chart.

Upon completing the dataset, a binary classification approach was adopted to categorize RSSI values into two classes: “Good” and “Bad”. This classification is based on a threshold of −50 dB. If the RSSI value is greater than −50 dB, it is classified as “Good”, indicating favorable signal strength. Conversely, values less than −50 dB are classified as “Bad”, indicating weaker signal strength. The resulting distribution of these classes is illustrated in a pie chart, as shown in Figure 7, where 43.7% of the measurements fall under the “Good” category, and 56.3% under “Bad”.

#### 3.2.7. Feature Extraction

Feature extraction was conducted to establish a comprehensive dataset for analysis. The experiment involved a rotating electric machine with four voltage levels (0 V, 25 V, 50 V, 100 V), collecting RSSI values both inside and outside the machine for each speed. The final dataset comprises 800 samples, derived from 100 measurements at each speed setting, for two locations. The dataset includes four main columns:

- t: Time (seconds);
- RSSI: Received Signal Strength Indicator (dB);
- U: Voltage level;
- IO: Measurement location indicator (0 for inside, 1 for outside).

These features form the foundation for further analysis, providing detailed information about signal propagation within the machine under different conditions.

#### 3.2.8. Feature Selection and Correlation Analysis

**Figure 8:**
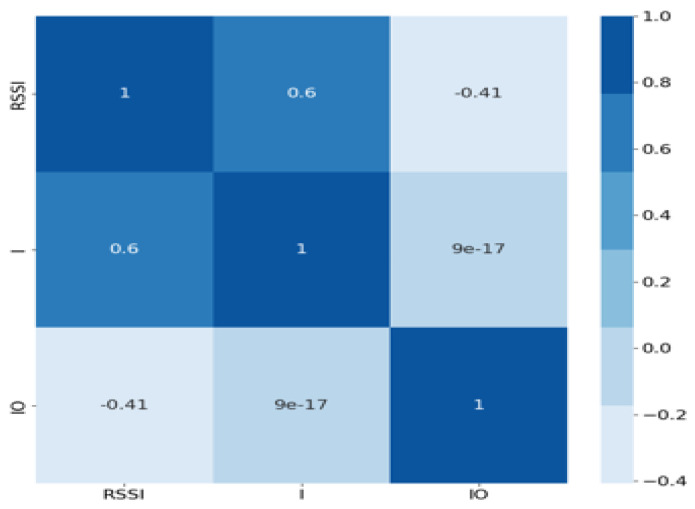
Correlation matrix.

Feature selection plays a crucial role in reducing data dimensionality and improving the efficiency of the classification framework. In the current analysis, a correlation matrix was constructed to identify relationships between the different features, which also aids in determining relevant features for the machine learning model. Figure 8 presents the correlation matrix, visually depicting the relationships between the variables.

Based on the correlation matrix, the following interpretations can be made:

- Diagonal Elements (1 s): Values on the diagonal are 1, representing perfect self-correlation.
- RSSI and Voltage (0.6): There is a moderate positive correlation between RSSI and voltage, suggesting that as RSSI increases, the voltage tends to increase as well.
- RSSI and Location Indicator (−0.41): There is a moderate negative correlation between RSSI and the location indicator, implying an inverse relationship between RSSI and whether the measurement was taken inside or outside the machine.
- Voltage and Location Indicator (9 × 10^−17^): The correlation value is approximately zero, indicating no significant linear relationship between these features.

For the dataset used in this analysis, only the RSSI, voltage (U), and location indicator (IO) variables will be considered, while the time variable will be excluded from further consideration. This correlation analysis enabled effective feature selection, focusing on the most relevant variables while removing redundant ones, ensuring a streamlined model for further analysis.

#### 3.2.9. Dataset Overview

The final dataset is well-structured with features suitable for machine learning classification tasks, aiming to provide insight into the communication quality within a rotating electric machine. The binary classification of RSSI, along with the feature extraction and selection, forms a strong foundation for training predictive models that can differentiate “Good” and “Bad” signal conditions with precision.

### 3.3. Model Selection and Rationale

This section describes the selection of machine learning models aimed at classifying RSSI data and accurately predicting communication quality within rotating electrical machines. Given the complex and often noisy environment associated with these machines, a variety of models were chosen to explore different ways of capturing patterns in RSSI data. The chosen models represent a spectrum of complexity and approach, allowing for a robust comparison to identify the most effective model for future applications.

The selected models include the following:

- Dummy Classifier: The Dummy Classifier was chosen as a baseline to set a minimum performance standard, making it easier to assess the added value of more sophisticated models. By using simple, rule-based predictions, the Dummy Classifier helps establish that any viable model should perform significantly better than random or frequency-based guesses.
- Gaussian Naive Bayes: Gaussian Naive Bayes (GNB) is included due to its effectiveness in handling continuous data and its simplicity, which makes it computationally efficient. This model’s assumption of feature independence provides a strong probabilistic baseline for classification tasks, allowing it to work well even with high-dimensional RSSI data. It is suitable for cases where certain RSSI features may be assumed to independently influence communication quality, which can yield a robust initial classification.
- Logistic Regression: Logistic Regression is widely used for binary classification and is well-suited for understanding the linear relationships between RSSI features and communication quality. By producing probabilities associated with class predictions, Logistic Regression offers valuable interpretability, making it useful for identifying direct correlations in RSSI data that may indicate varying communication quality levels.
- Quadratic Discriminant Analysis (QDA): QDA was chosen due to its ability to model complex, non-linear relationships by using a quadratic decision boundary. Since the distribution of RSSI data within rotating machines can exhibit non-linear patterns, QDA’s flexibility makes it particularly relevant. This model allows for unique covariance structures within each class, making it better suited for cases where the data cannot be easily separated by linear boundaries.
- Support Vector Machines (SVMs): SVM is a powerful model, especially for noisy, high-dimensional data, which is common in industrial environments with significant signal interference. The flexibility of SVM to employ different kernels, like the Polynomial and Radial Basis Function (RBF), makes it ideal for exploring both linear and non-linear decision boundaries. The RBF kernel, in particular, is beneficial for capturing complex relationships in RSSI data, as it allows for classification in an infinite-dimensional space. SVM was thus chosen for its robustness and capacity to handle the non-linear separations present in RSSI measurements from rotating machinery.

This diverse selection allows for a detailed evaluation of both simple and complex models, establishing whether simpler models like Gaussian Naive Bayes or Logistic Regression can achieve competitive performances or if more advanced techniques like QDA and SVM are required. By comparing these models, the objective is to identify the most challenging environment of rotating electrical machines.

### 3.4. Methodological Details

To enhance the reliability and robustness of the models, we also incorporated the following methodological steps:

#### 3.4.1. Parameter Tuning

Hyperparameter optimization was performed for each model using grid search combined with cross-validation. For example, for the Support Vector Machine (SVM), the optimal values for the regularization parameter and the kernel function (RBF) were chosen. Similarly, for models like Gaussian Naive Bayes, parameters were adjusted to better handle the high-dimensional nature of the RSSI data. The grid search allowed us to systematically explore parameter settings to improve model performance.

#### 3.4.2. Dataset Balancing

To mitigate the potential effects of class imbalance, especially in the context of wireless communication quality classification, we applied SMOTE (Synthetic Minority Over-sampling Technique) and random under-sampling techniques. These methods ensured that the models were trained on a balanced dataset, reducing the risk of bias and improving generalization.

#### 3.4.3. Model Training Settings

For model training, the dataset was split into training, validation, and testing sets. Stratified k-fold cross-validation was used to ensure that each fold maintained the proportion of the target classes, improving the reliability of performance metrics. The training process was conducted separately for each model, with tuning performed during the cross-validation process to avoid overfitting and ensure the best possible performance across different datasets.

### 3.5. Model Training

This section focuses on the third stage of the methodology, which involves training predictive models to classify RSSI data. The aim is to develop machine learning models capable of accurately predicting the communication quality within rotating electrical machines based on RSSI measurements. Several machine learning algorithms are applied and assessed to identify the most suitable one for this task.

The selected models include the following:

- Dummy Classifier;
- Gaussian Naive Bayes;
- Logistic Regression;
- Quadratic Discriminant Analysis;
- Support Vector Machines (SVM).

The training and evaluation of these models allow for a performance comparison, ultimately guiding the selection of the most effective model for predicting communication performance. This section explores the model selection process, training methods, and evaluation metrics used to determine the best model for future applications. This section provides a detailed description of each model.

#### 3.5.1. A Dummy Classifier

A Dummy Classifier is a simple baseline model used in machine learning to provide a reference point for evaluating the performance of more complex classifiers. It makes predictions using basic strategies without considering the input features. Common strategies include predicting the most frequent class, generating predictions that respect the class distribution, predicting classes uniformly at random, or always predicting a constant label. Dummy Classifiers are essential for establishing a baseline, ensuring that any meaningful model should perform better than these simplistic approaches. They are implemented in libraries like scikit-learn and are useful for highlighting the effectiveness of more sophisticated models [27].

#### 3.5.2. Gaussian Naive Bayes

The Gaussian Naive Bayes (GaussianNB) model is a probabilistic classifier based on Bayes’ Theorem, assuming that the features follow a Gaussian (normal) distribution. This model is particularly effective for continuous data. It calculates the mean and variance of each feature for every class during training. When making predictions, it uses these statistics to compute the probability that a given instance belongs to each class and selects the class with the highest probability. Gaussian Naive Bayes is widely used due to its simplicity and efficiency, especially in high-dimensional datasets. It is commonly applied in text classification, spam detection, and other scenarios where the assumption of feature independence holds reasonably well [28].

#### 3.5.3. Logistic Regression

Logistic Regression is a statistical model used for binary classification tasks, where the goal is to predict the probability that an instance belongs to one of two classes. It estimates the relationship between a dependent binary variable and one or more independent variables by using a logistic function, also known as the sigmoid function. This function maps any real-valued number into a value between 0 and 1, making it suitable for predicting probabilities.

In Logistic Regression, the output is interpreted as the probability of the dependent variable being 1 (e.g., success, yes, true) given the input features. The model is trained by finding the best-fitting parameters that maximize the likelihood of the observed data. Logistic Regression is widely used in various fields, including medical diagnosis, spam detection, and credit scoring, due to its simplicity and effectiveness [29].

#### 3.5.4. Quadratic Discriminant Analysis (QDA)

Quadratic Discriminant Analysis is a classification technique that extends Linear Discriminant Analysis (LDA) by allowing each class to have its own covariance matrix, rather than sharing a common one. This flexibility enables QDA to model more complex decision boundaries using quadratic functions, making it suitable for scenarios where the assumption of equal covariance matrices does not hold. By estimating the mean vector and covariance matrix for each class, QDA computes discriminant scores for observations and assigns them to the class with the highest score. While QDA can capture intricate relationships between features, it requires more parameters to be estimated, which can be challenging with small sample sizes. Despite being computationally intensive, QDA’s ability to model non-linear boundaries makes it valuable in fields like finance, biology, and marketing for tasks such as credit scoring, disease classification, and customer segmentation [30].

#### 3.5.5. Support Vector Machines (SVMs)

SVMs are powerful tools for classification and regression tasks. They work by finding the optimal hyperplane that separates data points of different classes. The choice of kernel function in SVMs is crucial for their performance. Here are explanations for the Polynomial (Poly) and Radial Basis Function (RBF) kernels [31]:

- Polynomial Kernel (Poly): This kernel represents the similarity of vectors in a feature space over polynomials of the original variables, allowing the algorithm to fit the maximum-margin hyperplane in a higher-dimensional space. The polynomial kernel is defined by the following equation:

*K(x, y) = (x × y + c)^d^* (1)

where *d* is the degree of the polynomial and *c* is a constant that can be adjusted. This kernel is useful for problems where the relationship between class labels and attributes is non-linear.

- Radial Basis Function (RBF) Kernel: Also known as the Gaussian kernel, the RBF kernel is one of the most popular and powerful kernels. It maps the input space into an infinite-dimensional space, making it capable of handling very complex relationships. The RBF kernel is defined by the following equation:

*K(x, y) = exp (−γ||x − y||*^2^*)* (2)

where *γ* is a parameter that defines the influence of a single training example. A higher *γ* value means a closer fit to the training data, while a lower value means a smoother decision boundary.

Both kernels enable SVMs to handle non-linear data by transforming it into a higher-dimensional space where a linear separator can be found.

### 3.6. Model Testing and Evaluating

The Model Testing and Evaluating phase is a crucial step in validating the performance of any machine learning model. This stage helps determine how well the model generalizes to provide insight into its reliability and effectiveness. In this study, the objective is to assess the accuracy of the trained model in predicting whether RSSI values indicate “Good” or “Bad” transmission quality within the rotating electric machine. Several evaluation metrics are used, each offering a different perspective on model performance.

#### 3.6.1. Accuracy

Accuracy is a measure of the overall effectiveness of the model and indicates the proportion of correctly classified instances out of the total instances. It is computed as the following equation [32]:

(3) $$\mathrm{Accuracy}=\frac{TP+TN}{TP+TN+FP+FN}$$

where:

- *TP* indicates True Positives—instances correctly classified as positive;
- *TN* indicates True Negatives—instances correctly classified as negative;
- *FP* indicates False Positives—instances incorrectly classified as positive;
- *FN* indicates False Negatives—instances incorrectly classified as negative.

#### 3.6.2. Precision

Precision measures the model’s ability to provide correct positive predictions and is defined as the following equation [33]:

(4) $$\mathrm{Precision}=\frac{TP}{TP+FP}$$

#### 3.6.3. Recall (Sensitivity or True Positive Rate)

Recall measures the model’s ability to identify all relevant positive instances. It is calculated as the following equation:

(5) $$\mathrm{Recall}=\frac{TP}{TP+FN}$$

#### 3.6.4. F1-Score

The *F1-score* is the harmonic mean of precision and recall, providing a balance between these two metrics. It is computed as the following equation [32]:

(6) $$F1-Score=2\times \frac{Precision\times Recall}{Precision+Recall}$$

#### 3.6.5. Confusion Matrix

A confusion matrix is a table that provides a detailed breakdown of prediction outcomes. It consists of four components: True Positives (*TP*), False Positives (*FP*), True Negatives (*TN*), and False Negatives (*FN*). It is presented as follows in Table 3 [32]:

#### 3.6.6. Support

Support is the number of true instances for each class in the dataset. It reflects the frequency of the class in the dataset. Support does not have a formula but is directly derived from the dataset as the count of true samples for a given class.

#### 3.6.7. Error Rate

The error rate measures the proportion of incorrect predictions made by the model, irrespective of the class.

(7) $$Error\;Rate=\frac{FP+FN}{TP+TN+FP+FN}$$

where TN is a True Negative (correctly classified as negative).

#### 3.6.8. Time (s)

This metric measures the computational time taken by the model to train and make predictions. It is important for evaluating runtime efficiency in real-world scenarios.

(8) $$Time=T_{end}-T_{start}$$

where:

- $T_{start}$ indicates the time when the process starts;
- $T_{end}$ indicates the time when the process ends.

#### 3.6.9. The Receiver Operating Characteristic

The Receiver Operating Characteristic (ROC) curve is a graphical representation used to evaluate the performance of binary classification models. It plots the True Positive Rate (*TPR*), also called Sensitivity, against the False Positive Rate (*FPR*) at various threshold settings [32].

- True Positive Rate (*TPR*) or Sensitivity: This measures the proportion of actual positive cases (e.g., “Good” RSSI) that are correctly identified by the model [32].

(9) $$\mathrm{TPR}=\frac{True\;Positives\;(TP)}{True\;Positives\left(TP\right)+False\;Negatives\;(FN)}$$

- High *TPR* means the model is correctly identifying most of the positive instances.
- False Positive Rate (*FPR*): This indicates the proportion of actual negative cases (e.g., “Bad” RSSI) that are incorrectly classified as positive by the model.

(10) $$\mathrm{FPR}=\frac{False\;Positives\;(FP)}{False\;Positives\left(FP\right)+True\;Negatives\;(TN)}$$

- A low *FPR* means that the model makes fewer incorrect positive classifications.

The Area Under the Curve (AUC) represents the degree of separability achieved by the model, showing how well the model distinguishes between the two classes. AUC values range from 0.5 to 1. A value of 0.5 means the model performs no better than random chance. A value close to 1 indicates a near-perfect model. The higher the AUC, the better the model is at predicting true positives while minimizing false positives.

The ROC curve itself is a plot of *TPR* vs. *FPR*. The point (0,1) on the ROC curve represents perfect classification, where *TPR* is 1 (all positives are correctly identified), and *FPR* is 0 (no negatives are incorrectly classified as positives). The closer the curve follows the top-left border of the plot, the better the model’s performance. The ROC curve helps to visualize the performance of a classifier across all threshold levels, providing a more complete picture than just accuracy or confusion matrices. It is useful in selecting the optimal threshold where *TPR* is maximized while minimizing FPR. In summary, ROC and AUC give a robust understanding of the trade-offs between true positive and false positive rates, which is essential when evaluating the effectiveness of a classifier, especially in applications like real-time wireless communication performance, where minimizing errors is critical.

The Model Testing and Evaluating step is critical for validating the classifier’s capability to distinguish between “Good” and “Bad” RSSI values, ensuring the communication quality inside the rotating electric machine is monitored accurately. Using metrics like precision, recall, *F1-score*, accuracy, support, time, error, and a confusion matrix, allows for a comprehensive evaluation of the model, making it possible to understand and improve its robustness, especially in complex environments where machine reliability and control are essential. This helps in developing a highly reliable communication monitoring framework, essential for maintenance and predictive failure detection in rotating electric machines.

## 4. Results and Discussions

The dataset used in this study was prepared to represent realistic conditions of wireless communication within rotating electric machines. To develop robust predictive models, the dataset was divided into training and test sets, with cross-validation applied to mitigate overfitting and ensure generalizability. Each machine learning model underwent hyperparameter tuning through grid search, particularly for parameters such as gamma in the SVM model (see Equation (2)). This optimization ensured that each model’s performance, as displayed in the confusion matrices and summarized in Table 3, reflects optimal parameter settings.

This study implements five machine learning models on the dataset, utilizing Kaggle as the primary tool for analysis. The dataset was divided into training and test sets, with cross-validation applied to enhance the robustness of the models and prevent overfitting. For each model, grid search was used to optimize key parameters (e.g., gamma for SVM-RBF, as shown in Equation (2)). These optimization steps ensure that the reported metrics in Table 3 are reflective of each model’s best achievable performance. The evaluation of these models is crucial in determining their performance in real-time wireless communication within rotating electric machines.

**Figure 9:**
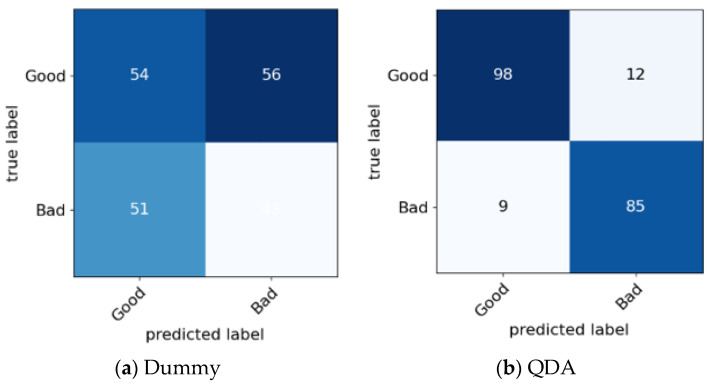

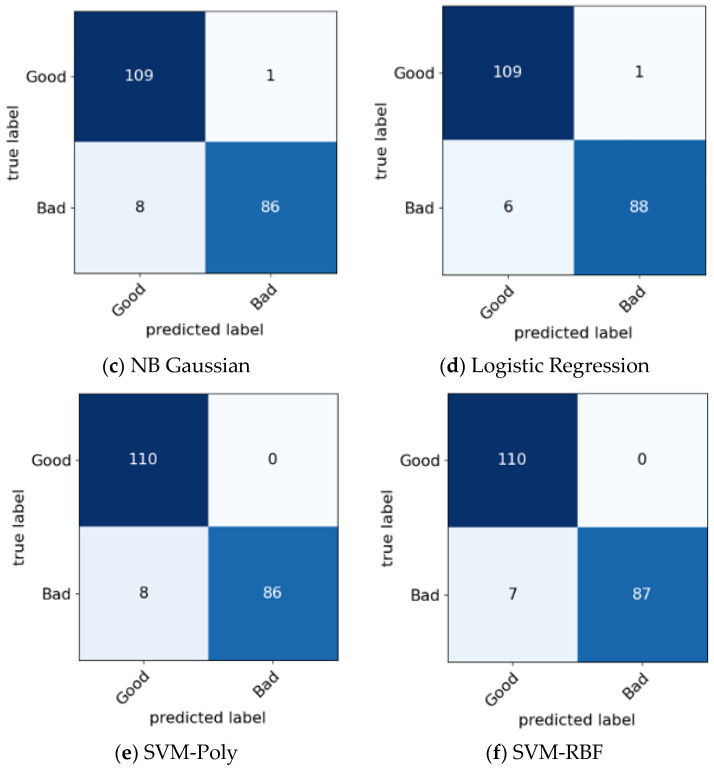
Confusion matrices obtained using different models.

As the first step, the performance of each model is illustrated using confusion matrices, displayed in Figure 9.

This visual representation aids in understanding the classification accuracy of the models.

Upon examining the confusion matrices, the SVM-RBF model demonstrated minimal misclassification, with only seven false positives and zero false negatives. The false positives may arise from subtle variations in RSSI values that fall close to the decision threshold, leading to misclassifications of “Bad” values as “Good”. The model’s ability to correctly classify the majority of instances is reflected in its high recall for both classes. The low false positive rate suggests the model’s effectiveness in avoiding false alarms in the dynamic environment of rotating machines.

Subsequently, Table 4 summarizes the key metrics for each model, including precision, recall, F1-score, accuracy, support, time, and error.

The findings indicate that the Support Vector Machine with a Radial Basis Function kernel (SVM-RBF) outperforms the other models. The SVM-RBF model showcases remarkable results, with precision values of 0.95 for the “Bad” class and 0.99 for the “Good” class, underscoring its strong classification capabilities while maintaining a low rate of false positives. Furthermore, the recall rates of 0.99 for the “Bad” class and 0.93 for the “Good” class highlight its effectiveness in capturing a majority of true instances for both classes. The F1-score, which balances precision and recall, reached 0.97, reaffirming the model’s robustness across both categories. Overall, an accuracy of 97% emphasizes the model’s reliability in predicting true RSSI classes.

Comparatively, while models like Logistic Regression and Gaussian Naive Bayes also delivered commendable results, they did not match the precision and F1-scores achieved by SVM-RBF. Despite strong recall metrics, the comprehensive performance of SVM-RBF in terms of precision, recall, and *F1-score* establishes it as the most suitable choice for this classification task.

The confusion matrix provides further confirmation of the SVM-RBF model’s superiority, revealing 110 true positives, 87 true negatives, zero false negatives, and only seven false positives. This minimal misclassification rate highlights the model’s reliability in detecting “Good” and “Bad” RSSI values, particularly crucial in the dynamic environment of rotating machines.

**Figure 10:**
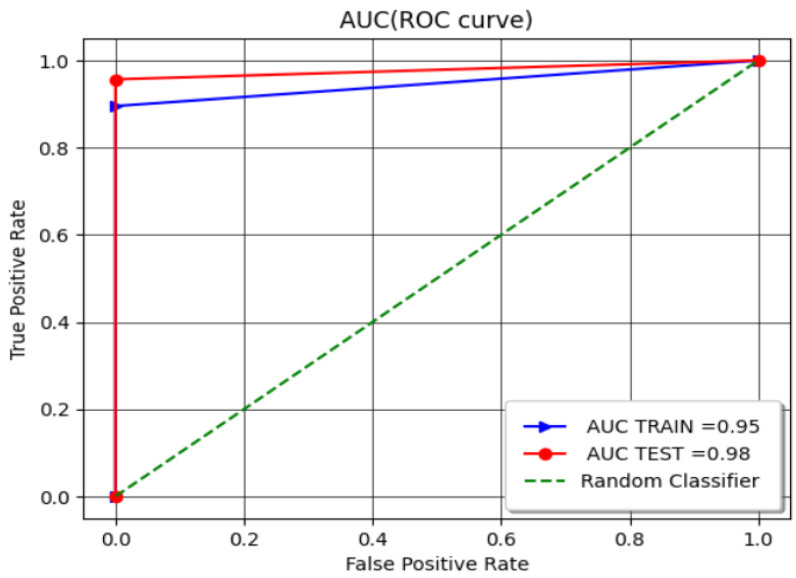
The ROC curve of the SVM-RBF model.

The ROC curve was generated to further evaluate the model’s performance. The ROC curve plots the true positive rate against the false positive rate at various threshold settings. A model with a ROC curve that approaches the top-left corner indicates better performance. Figure 10 presents the ROC curve.

The AUC for the test set is slightly higher than that of the training set, which can be attributed to several factors, including the relatively small size of the test set and random variations in the data. Additionally, the use of regularization in the model prevents overfitting, which can lead to improved generalization on the test set. Moreover, noise or outliers present in the training data, which the model struggles to fit perfectly, may have contributed to the observed difference in AUC values.

For the SVM-RBF model, the area under the ROC curve (AUC) was found to be very high, demonstrating its excellent ability to distinguish between the “Good” and “Bad” RSSI values. This comprehensive analysis underscores the model’s capability in providing reliable communication performance evaluations in complex machine environments.

The SVM-RBF model outperformed other models primarily due to the ability of the RBF kernel to handle non-linear decision boundaries. The RBF kernel is particularly well-suited for problems where the classes are not linearly separable, as it maps input data to a higher-dimensional space, where a linear decision boundary can be more easily established. In our case, the RSSI values exhibit complex patterns that benefit from the non-linear transformation provided by the RBF kernel. This leads to superior classification performance, as reflected in the higher precision, recall, and F1-scores achieved by the SVM-RBF model. In summary, the SVM-RBF model emerges as the most effective choice for evaluating communication performance based on RSSI measurements, characterized by high precision, recall, F1-score, and minimal classification errors.

The superior performance of the SVM-RBF model can be attributed to its ability to handle non-linear decision boundaries, which is critical in environments with complex patterns in the RSSI data. Unlike Logistic Regression, which assumes linear separability, RBF kernel maps data into a higher-dimensional space, enabling more effective separation of non-linear patterns. Similarly, while QDA is well-suited for data with distinct Gaussian distributions, the RSSI data collected in our experiments exhibited substantial variability due to environmental factors such as reflections, metallic surfaces, and machine vibrations. These factors introduce randomness that challenges QDA’s assumption of Gaussian distributions for each class.

In terms of runtime, the SVM-RBF model delivered comparable results to other models, with certain algorithms demonstrating higher simulation times. Despite this, the SVM-RBF model consistently outperformed others in error reduction, confirming its robustness for classification tasks. Additionally, the support parameter yielded similar values across all models, further underscoring the reliability and effectiveness of SVM-RBF in managing complex and variable data environments.

The RBF kernel excels in capturing intricate patterns by creating flexible decision boundaries that adapt to the underlying data structure. For instance, overlapping RSSI values caused by signal interference are more accurately classified by the SVM-RBF model due to its non-linear transformation capabilities. This advantage is supported by the significantly higher precision, recall, and F1-scores observed for SVM-RBF compared to Logistic Regression and QDA.

Furthermore, the ability of the RBF kernel to generalize effectively on unseen data enhances its robustness in evaluating communication performance under diverse conditions. These findings emphasize the importance of selecting a model architecture that aligns with the specific characteristics of the dataset and the environmental factors influencing it.

A limitation of the current study is the reliance on a single test stand and hardware setup. While this controlled environment enables initial observations, it may restrict the generalizability of the findings to other setups. Future research will include testing in diverse environments and with varied hardware configurations to evaluate the robustness of the proposed model. These steps are essential for verifying the model’s adaptability and ensuring its applicability in broader contexts.

The randomness inherent in RSSI data is primarily due to environmental and system-induced variations, such as reflections, metallic surfaces, and the rotation of the machine. These variations can cause fluctuations in the measured RSSI values, making their classification and interpretation challenging. By incorporating artificial intelligence and machine learning (AI/ML) techniques, this study effectively classifies these random patterns, extracting meaningful insights about communication performance. In future work, additional parameters, such as rotor temperature, will be integrated with RSSI measurements. This combination could enhance the monitoring system by verifying whether detected temperature values are accurate or influenced by poor communication conditions, such as low RSSI levels. This integration could enable proactive interventions, such as stopping the motor when critical temperature thresholds are reached, thereby improving system reliability and safety.

Furthermore, future work will explore predictive modeling for both RSSI and temperature values, aiming to forecast potential failures and optimize monitoring systems. This stepwise approach not only addresses the inherent randomness in RSSI data but also lays the groundwork for broader and more impactful applications in evaluating wireless communication within rotating electrical machines.

The proposed architecture is highly significant for industrial applications, particularly in the monitoring and control of rotating electric machines. By leveraging real-time RSSI measurements and advanced machine learning algorithms, this system offers a robust solution for detecting and predicting anomalies, ensuring optimal operational efficiency and safety.

In industrial environments, such as automotive engines or aircraft turbines, any failure in communication or machine components can have catastrophic consequences. The integration of machine learning not only facilitates the classification of RSSI data to evaluate communication performance but also enables the prediction of potential anomalies before they manifest into critical issues. For instance, in the case of an aircraft engine, a sudden increase in rotor temperature combined could indicate an impending failure. By detecting such conditions early, the system could alert the pilot, allowing for preventive action and avoiding potential disasters.

This approach mirrors systems like the Tire Pressure Monitoring System (TPMS), which monitors tire pressure and temperature in real time to alert drivers of potential risks. Similarly, the proposed methodology provides a foundation for predictive maintenance by continuously monitoring key parameters and issuing alerts when thresholds are exceeded. For example, in the automotive industry, this system could alert drivers of an abnormal rise in rotor temperature or communication degradation in electric motors, ensuring timely interventions. In aviation, where safety margins are critical, the system could play a pivotal role in monitoring aircraft engine health, avoiding malfunctions mid-flight.

By utilizing real-world measurements and combining them with AI-driven predictions, the system offers a dual benefit:

- Real-time Monitoring: Immediate assessment of communication quality and machine conditions, enabling on-the-spot decision-making.
- Predictive Insights: Proactive anomaly detection to prevent failures, reduce downtime, and enhance overall safety and reliability in industrial operations.

This architecture provides a transformative solution for applications ranging from manufacturing plants to transportation systems. Its scalability, versatility, and alignment with Industry 4.0 principles make it a valuable tool for predictive maintenance in modern industrial systems.

## 5. Conclusions

This paper presents an innovative methodology for evaluating communication performance within rotating electric machines using Received Signal Strength Indication (RSSI) measurements and artificial intelligence. The proposed approach offers valuable insights into the propagation behavior of wireless signals in the challenging and dynamic environment inside a rotating electric machine. By focusing on RSSI as a key parameter, the methodology enables the real-time assessment of signal quality, which is essential for addressing issues like reflections, metallic surfaces, and the effects of machine rotation on communication reliability.

The methodology consists of several crucial stages, including data collection via an advanced hardware setup, data preprocessing with binary classification, feature extraction, model training, and the final testing and evaluation of several machine learning models. The results highlight the capability of artificial intelligence in effectively classifying RSSI data into categories based on a predetermined threshold. Multiple models, such as the Dummy Classifier, Gaussian Naive Bayes, Logistic Regression, Quadratic Discriminant Analysis (QDA), and Support Vector Machines (SVM), were evaluated. Among these, the SVM model demonstrated the highest performance on the dataset, with superior precision, recall, and F1-scores. Specifically, the SVM model with a Radial Basis Function (RBF) kernel achieved an accuracy of 97%, supported by precision and recall values nearing 1.0. This indicates that the SVM model is well-suited to handle the complexities of this environment. The confusion matrix further confirms the model’s strength, showing minimal data misclassification, which solidifies SVM as the most reliable choice for this type of analysis.

The contribution of this work lies in the effective combination of real-time RSSI measurements and artificial intelligence to monitor and control the quality of wireless communication within rotating electric machines. This approach addresses critical communication challenges in complex industrial settings, opening avenues for more advanced, automated monitoring systems. These systems will be capable of ensuring consistent communication performances, even in environments subject to high levels of electromagnetic interference. The success of this methodology points to the possibility of developing future systems that autonomously adjust signal parameters to optimize communication performance and detect issues before they escalate into critical failures.

While the current methodology focuses on RSSI, the incorporation of other wireless communication metrics, such as Signal-to-Noise Ratio (SNR), could offer a more comprehensive understanding of communication quality in these environments. The inclusion of SNR in future work could further enhance the analysis, providing additional insights into the interference and noise levels affecting signal propagation, which are crucial factors for ensuring reliable communication in industrial applications.

This study provides an initial evaluation of RSSI-based communication performance within a rotating machine but is limited to a controlled setup with specific experimental conditions. Future research will focus on exploring the influence of the machine’s internal environment and rotational dynamics on link reliability. Expanding this work to include ambient Wi-Fi signal measurements across all 2.4 GHz channels, both inside and outside the machine, would provide additional insights into interference levels and their effect on communication stability. In addition, quantifying the impact of metallic surfaces and rotational dynamics on RSSI through simulations and experimental analysis will enhance the understanding of signal propagation challenges. Simulations will model varying surface properties and rotation speeds, while experiments will systematically vary these parameters to evaluate their influence on signal strength, attenuation, and interference patterns. Statistical analyses will further quantify the correlations between these factors and RSSI variability. This will allow for a comprehensive assessment of how these environmental factors impact communication reliability. Furthermore, validating the methodology across different hardware setups and environments will help assess its robustness and generalizability. For instance, experiments involving diverse machine types, environmental conditions, and operational parameters will be conducted to demonstrate the system’s adaptability.

Future work will also explore deploying this methodology in real-world industrial scenarios, such as manufacturing plants, automotive systems, and aircraft turbines. This will involve developing a more advanced system architecture that integrates transfer learning techniques, allowing models to adapt quickly to new configurations and minimize retraining efforts. The integration of additional sensory data, such as rotor temperature and vibration metrics, could further enrich the analysis, enabling predictive maintenance and early fault detection.

The integration of artificial intelligence into the control and monitoring of rotating electric machines opens exciting future possibilities. Advanced AI models could be utilized for predictive maintenance, real-time fault detection, and dynamic optimization of communication parameters. Future systems would go beyond monitoring the communication link, actively improving machine performance by detecting potential disruptions or inefficiencies early on. This would pave the way for more reliable, efficient operations in industrial settings, revolutionizing how electric machines are managed and controlled.

## Figures and Tables

**Table 1:** Comparison of some related works.

Methodology	Key Features	Strengths	Limitations
Wireless Sensor-Based Monitoring [6]	Wireless sensors collect data (e.g., temperature, vibration) for real-time monitoring.	Reduces wiring complexity; enables real-time monitoring in industrial settings.	Does not evaluate communication quality (e.g., RSSI performance) under varying machine conditions.
Classical Models (e.g., State–Space, etc.) [12,13,14]	Analytical models using mathematical equations to predict machine performance and diagnose faults.	Provide foundational insights into machine dynamics; well-established for theoretical analysis.	Require manual calculations; lack adaptability for real-time applications in dynamic environments.
RSSI-Based Performance Assessment [15,16]	RSSI used to measure signal quality in telecommunication systems and optimize network performance.	Effective for environments with obstructions or interference; widely used in WSNs and IoT applications.	Limited application in the context of rotating electrical machines.
AI in Communication Networks [17,20]	Deep learning applied for predicting network performance degradation and optimizing infrastructure.	Provides real-time adaptive solutions; uses signal quality metrics like RSSI for predictions.	Primarily focuses on general communication networks, not industrial applications.
AI in TPMS [21,22]	AI models enhance monitoring systems, improving predictive maintenance for tires.	Demonstrates ML effectiveness in dynamic, real-time systems; reduces failure risks.	Focuses on TPMS, not applicable to communication in electrical machines.
ML and IoT for Industry 4.0 [25]	Combines ML and IoT for predictive maintenance, security, and process control in industrial settings.	Enhances resource management, operational safety, and scalability; aligns with Industry 4.0 objectives.	General focus on manufacturing applications; limited focus on rotating electrical machine environments.
Proposed Methodology (RSSI + ML in Rotating Machines)	Combines RSSI measurements with machine learning models to classify communication quality in rotating electrical machines.	Real-time monitoring of communication performance using RSSI; adaptive and scalable; improves fault detection and performance evaluation.	Complexity in integrating real-time signal processing with machine learning models in industrial environments.

**Table 2:** Induction machine model parameters.

Parameter	Value
Power	2.27 kW
Rated Voltage	110 V
Number of Poles	2
Speed	1500 rpm
Frequency	50 Hz
Number of Stator Slots	24
Number of Rotor Slots	28

**Table 3:** Confusion Matrix.

	Predicted Positive	Predicted Negative
Actual Positive	True Positive (*TP*)	False Negative (*FN*)
Actual Negative	False Positive (*FP*)	True Negative (*TN*)

**Table 4:** Evaluation metrics for different machine learning models.

Model	Evaluating Metrics	C0	C1	Accuracy	Support	Time (s)	Error
Dummy	Precision	0.51	0.49	0.47			
	Recall	0.49	0.46		88.0	0.002	0.431
	F1-score	0.50	0.45				
QDA	Precision	0.92	0.88	0.90			
	Recall	0.89	0.90		88.0	0.007	0.107
	F1-score	0.90	0.89				
Logistic Regression	Precision	0.95	0.99	0.96			
	Recall	0.99	0.94		88.0	0.438	0.068
	F1-score	0.97	0.96				
NB Gaussian	Precision	0.93	0.99	0.95			
	Recall	0.99	0.91		88.0	0.005	0.073
	F1-score	0.96	0.95				
SVM-Poly	Precision	0.93	0.99	0.96			
	Recall	0.99	0.91		88.0	0.106	0.049
	F1-score	0.96	0.96				
SVM-RBF	Precision	0.95	0.99				
	Recall	0.99	0.93	0.97	88.0	0.022	0.001
	F1-score	0.97	0.97				

## Data Availability

The data supporting the findings of this study are not publicly available due to confidentiality restrictions. The RSSI measurements used in this research were collected as part of a proprietary experimental setup, and access to the data is restricted to protect the privacy and intellectual property associated with the study.

## References

[1] Liu P., Liu X., Zhang Y., Hu B., Liang Z. Comparison of Different Simulating Calculation Methods for Electric Fields Along the Stator End-winding of HV Rotating Machine. Proceedings of the 2020 IEEE Electrical Insulation Conference (EIC).

[2] Kudelina K., Raja H.A., Autsou S., Naseer M.U., Vaimann T., Kallaste A., Pomamacki R., Hyunh V.K. Preliminary Analysis of Mechanical Bearing Faults for Predictive Maintenance of Electrical Machines. Proceedings of the 2023 IEEE 14th International Symposium on Diagnostics for Electrical Machines, Power Electronics and Drives (SDEMPED).

[3] Roger D., Ninet O. Vector control of dual stator winding induction machine: A new technique to neutralize effects of rotor time constant variations. Proceedings of the IEEE International Electric Machines and Drives Conference, IEMDC’03.

[4] Ben Brahim S., Dardouri S., Hammami A., Bouallegue R., David J., Vuong T.-H. (2024). The Impact of an Electric Machine Body on EM Wave Propagation in RTMS. Machines.

[5] Ben Brahim S., Bouallegue R., David J., Vuong T.H. (2019). Study and improvement in the radio communication quality for rotating electrical machine. Electr. Eng..

[6] Ben Brahim S., Bouallegue R., David J., Vuong T.H., David M. (2017). A Wireless On-line Temperature Monitoring System for Rotating Electrical Machine. Wirel. Pers. Commun..

[7] Luo Q., Peng Y., Li J., Peng X. (2016). RSSI-Based Localization Through Uncertain Data Mapping for Wireless Sensor Networks. IEEE Sens. J..

[8] Hossen M.S., Kamal M.K.B., Rahman M.S. Consistency analysis of RSSI measurement for distance estimation of Wireless Sensor nodes. Proceedings of the 2012 15th International Conference on Computer and Information Technology (ICCIT).

[9] Baeza-Yates R., Fayyad U.M. (2024). Responsible AI: An Urgent Mandate. IEEE Intell. Syst..

[10] Adams S., Taylor M., Crofford C., Harper S., Batchelor W., Headley W.C. Exploring Explainable AI Techniques for Radio Frequency Machine Learning. Proceedings of the 2024 IEEE International Conference on Machine Learning for Communication and Networking (ICMLCN).

[11] Geigel A. (2023). Machine learning AI systems and the virtue of inventiveness. AI Ethics.

[12] Dias C.G., da Silva L.C., Chabu I.E. (2019). Fuzzy-Based Statistical Feature Extraction for Detecting Broken Rotor Bars in Line-Fed and Inverter-Fed Induction Motors. Energies.

[13] Tarchała G., Wolkiewicz M. (2019). Performance of the Stator Winding Fault Diagnosis in Sensorless Induction Motor Drive. Energies.

[14] Frosini L. (2020). Novel Diagnostic Techniques for Rotating Electrical Machines—A Review. Energies.

[15] Kumar V., Arablouei R. (2022). Self-Localization of IoT Devices Using Noisy Anchor Positions and RSSI Measurements. Wirel. Pers. Commun..

[16] Rahman A.A. RSSI-Guided Cluster Head Selection for Optimal Optimization in IoT-Enabled WSNs. Proceedings of the 2023 IEEE 8th International Conference on Software Engineering and Computer Systems (ICSECS).

[17] Rawas S. (2024). AI: The future of humanity. Discov. Artif. Intell..

[18] Knafo D. (2024). Artificial Intelligence on The Couch. Staying Human Post-AI. Am J. Psychoanal..

[19] Humm B.G., Archer P., Bense H., Bernier C., Goetz C., Hoppe T., Schumann F., Siegel M., Wenning R., Zender A. (2023). New directions for applied knowledge-based AI and machine learning. Inform. Spektrum.

[20] Nancharaiah B., Ravi K.C., Srivastava A.K., Arunkumar K., Siddiqui S.T., Arun M.R. (2023). Analysis of Data Science and AI-enabled 6G Wireless Communication Networks. Radioelectron. Commun. Syst..

[21] Zainal Abidin A.N.S., Jamaludin A.S., Nasir A., Sufian A.H., Rosli A.M., Aziz R.A., Ismail Z., Iqbal A.K.M.A., Ahmed I. (2024). Current Developments and Future Prospects in Vehicle Tire Technologies: A Review. Intelligent Manufacturing and Mechatronics. iM3F 2023.

[22] Ikeda Y., Kato A., Kohjiya S., Nakajima Y. (2024). Pneumatic Tire Technology. Rubber Science.

[23] Zhao Q., Fu H., Zhang Y., Gang X., Tan D. (2021). Research on load prediction model construction method of the tire condition monitoring system. J. Braz. Soc. Mech. Sci. Eng..

[24] Nagarajan J., Mansourian P., Shahid M.A., Jaekel A., Saini I., Zhang N., Kneppers M. (2023). Machine Learning based intrusion detection systems for connected autonomous vehicles: A survey. Peer Peer Netw. Appl..

[25] Angelopoulos A., Michailidis E.T., Nomikos N., Trakadas P., Hatziefremidis A., Voliotis S., Zahariadis T. (2020). Tackling Faults in the Industry 4.0 Era—A Survey of Machine-Learning Solutions and Key Aspects. Sensors.

[26] Pham P.P. (2005). Comprehensive Analysis of the IEEE 802.11. Mobile Netw. Appl..

[27] Liu Z., Chen G., Li Z., Kang Y., Qu S., Jiang C. (2023). PSDC: A Prototype-Based Shared-Dummy Classifier Model for Open-Set Domain Adaptation. IEEE Trans. Cybern..

[28] Testas A. (2023). Naive Bayes Classification with Pandas, Scikit-Learn, and PySpark. Distributed Machine Learning with PySpark.

[29] Geng Y., Li Q., Yang G., Qiu W. (2024). Logistic Regression. Practical Machine Learning Illustrated with KNIME.

[30] Härdle W.K., Simar L., Fengler M.R. (2024). Discriminant Analysis. Applied Multivariate Statistical Analysis.

[31] Kecman V., Wang L. (2005). Support Vector Machines—An Introduction. Support Vector Machines: Theory and Applications.

[32] Ragab D.A., Sharkas M., Attallah O. (2019). Breast cancer diagnosis using an efficient cad system based on multiple classifiers. Diagnostics.

[33] Novaković J.D., Veljović A., Ilić S.S., Papić Ž., Milica T. (2017). Evaluation of classification models in machine learning. Theory Appl. Math. Amp. Comput. Sci..

